# Systematic Review of Nasal Endoscopy Scores in Cystic Fibrosis Patients Treated With Cystic Fibrosis Transmembrane Conductance Regulator Modulators

**DOI:** 10.1002/ppul.71601

**Published:** 2026-04-01

**Authors:** Rebecca Gallardo, Bruna Santos Silva, Lucas Sousa Salgado, José Dirceu Ribeiro, Eulalia Sakano

**Affiliations:** ^1^ Departamento de Otorrinolaringologia e Cirurgia de Cabeça e Pescoço Universidade Estadual de Campinas (UNICAMP) Campinas São Paulo Brasil; ^2^ University of Taubaté (UNITAU) Caraguatatuba São Paulo Brazil; ^3^ Departamento de Pediatria Universidade Estadual de Campinas (UNICAMP) Campinas São Paulo Brasil

**Keywords:** CFTR modulators, chronic rhinosinusitis, cystic fibrosis, Lund‐Kennedy score, nasal endoscopy, triple therapy

## Abstract

**Introduction:**

Cystic fibrosis (CF) is a severe genetic disorder caused by pathogenic variants in the Cystic Fibrosis Transmembrane Conductance Regulator (*CFTR*) gene, leading to multisystem complications including chronic rhinosinusitis and nasal polyposis. Recent advances in CFTR modulator therapies have revolutionized systemic disease control, but their impact on sinonasal disease remains less explored.

**Methods:**

A systematic review was conducted following Preferred Reporting Items for Systematic Reviews and Meta‐Analyses (PRISMA) guidelines and registered in International Prospective Register of Systematic Reviews (PROSPERO, registration number CRD42025632498). Searches were performed across PubMed/MEDLINE, Cochrane Library, and Embase through March 2025. Studies reporting nasal endoscopic outcomes using validated scoring systems before and after CFTR modulator therapy were included.

**Results:**

Out of 232 identified records, 10 studies met inclusion criteria, representing populations from six countries. Most studies assessed triple therapy (Elexacaftor/Tezacaftor/Ivacaftor). Endoscopic scores, including the Modified Lund‐Kennedy scale, showed significant reductions in nasal polyps, mucosal edema, and discharge. Pediatric and adult groups benefited alike, with triple therapy proving more effective than dual or monotherapy. Secondary outcomes included improved pulmonary function, weight gain, and olfactory recovery.

**Conclusion:**

This review demonstrates that CFTR modulators provide significant benefits for sinonasal disease in CF, reinforcing their role as a comprehensive therapeutic approach addressing both upper airway and systemic disease burdens.

## 1 Background

Cystic fibrosis (OMIM: n°. 219700) is a genetic and life‐threatening disorder that affects more than 80,000 individuals worldwide, having a significant impact on the quality of life of those affected [1]. This autosomal recessive condition is caused by mutations in the gene responsible for encoding the cystic fibrosis transmembrane conductance regulator protein, namely *CFTR* gene [2, 3]. This protein regulates the transport of chloride and bicarbonate ions across epithelial surfaces. When pathogenic genetic variants are present, ion transport becomes impaired, resulting in abnormally thick and viscous mucus in various organs of the body [2, 4].

The disease may cause dysfunctions in multiple systems, including the gastrointestinal tract, pulmonary system, exocrine pancreas, and upper airways. In the upper respiratory tract, one of the consequences of excessive mucus viscosity is chronic inflammation, which can progress to chronic rhinosinusitis [2, 5]. Chronic rhinosinusitis is the most common upper airway disorder observed in individuals with cystic fibrosis, affecting nearly one hundred percent of patients, with or without nasal polyps [6]. It typically begins early in life; however, the subjective clinical symptoms may vary among children and adults depending on the extent of sinonasal disease [7].

It is estimated that more than 90,000 people worldwide live with cystic fibrosis, mainly in North America, Australia, and Western Europe [8]. In the general population, the prevalence of chronic rhinosinusitis ranges from 3% to 6% when symptoms are confirmed by endoscopic or imaging findings. Among patients with cystic fibrosis, almost all show signs of sinonasal inflammation on imaging or endoscopy, but only 20% to 60% report symptoms. This discrepancy may be related to patients' adaptation to living with a chronic disease or to more severe pulmonary symptoms, although there is no definitive explanation [9, 10].

According to the European Position Paper on Rhinosinusitis and Nasal Polyps [11], rhinosinusitis is defined as inflammation of the nasal and paranasal sinus mucosa, diagnosed when two or more of the following symptoms are present: nasal obstruction, anterior or posterior nasal discharge, facial pain or pressure, and reduced or lost sense of smell. These symptoms may be associated with endoscopic findings such as nasal polyps, purulent discharge in the middle meatus, or mucosal edema, in addition to imaging changes. The presence of nasal polyps defines a subgroup of chronic rhinosinusitis. In cystic fibrosis patients, extensive radiological changes in the paranasal sinuses are common even without clinical symptoms or endoscopic findings, making it difficult to correlate imaging with the patient's clinical condition [12].

Furthermore, up to fifty percent of individuals with cystic fibrosis develop nasal polyposis [12], which can be identified through objective diagnostic methods such as nasal endoscopy. Several endoscopic nasal polyp scoring systems have been described in the scientific literature [13, 14]. Among the most widely used are the Modified Lund‐Kennedy score and the Nasal Polyp score [14, 15]. The Modified Lund‐Kennedy score assesses three parameters: presence of polyps, degree of mucosal inflammation, and presence of nasal discharge [16]. The Nasal Polyp score classifies polyps based on size as follows: 0 – no polyps present, 1 – small polyps located in the middle nasal meatus that do not reach below the inferior border of the middle turbinate, 2 – polyps extending below the inferior border of the middle turbinate, 3 – large polyps reaching the lower border of the inferior turbinate or located medial to the middle turbinate, and 4 – large polyps causing complete obstruction of the inferior nasal cavity [17].

In recent years, the therapeutic approach for cystic fibrosis has been transformed by the development of medications that modulate the function of the CFTR protein [18]. The United States Food and Drug Administration has approved four such modulators for individuals with specific genetic pathogenic variants: Ivacaftor, Lumacaftor/Ivacaftor, Tezacaftor/Ivacaftor, and the most recent and comprehensive triple‐combination therapy Elexacaftor/Tezacaftor/Ivacaftor [19]. These modulators, particularly the triple combination therapy, have not only significantly reduced the systemic burden of disease in individuals with cystic fibrosis, but also contributed to improved control of chronic rhinosinusitis. Studies have reported improvements in endoscopic scores, including reductions in nasal polyp size and mucosal inflammation [13].

Although a previous meta‐analysis has explored the functional and radiological outcomes of CFTR protein modulators in relation to sinus disease [19], to date there is no systematic review in the scientific databases that synthesizes findings specifically from nasal endoscopy scoring systems in the context of this new therapeutic era. In this regard, the present study aims to contribute to scientific knowledge by compiling and analyzing available data on nasal endoscopy findings in individuals with cystic fibrosis treated with protein modulators, supporting improved clinical management and enhancing the quality of life for this population.

## 2 Methods

The protocol for this review is registered in the International Prospective Register of Systematic Reviews (PROSPERO) under registration number CRD42025632498. The presentation of the results follows the Preferred Reporting Items for Systematic Reviews and Meta‐Analyses (PRISMA) guidelines. This review aimed to synthesize endoscopic nasal findings and their evaluation in the context of CFTR modulator therapies.

A comprehensive literature search was conducted across PubMed/MEDLINE, Cochrane Library, and Embase, covering all studies available up to March 2025. The following search strategy was applied:

“cystic fibrosis” AND (“elexacaftor/tezacaftor/ivacaftor” OR “triple therapy” OR ivacaftor OR kalydeco OR “CFTR modulators”) AND (“nasal endoscopic findings” OR “rigid nasal endoscopy” OR “Lund‐Kennedy score” OR nasal polyp OR sinonasal outcomes OR “chronic rhinosinusitis”)

### 2.1 Study Selection

All retrieved citations were exported and managed using Zotero. Two independent reviewers (R.G.A. and B.S.) screened the titles and abstracts for relevance based on predefined inclusion and exclusion criteria. Any disagreements were resolved through consensus with a third reviewer (E.S.). Duplicate records were removed prior to screening.

No restrictions were placed on publication year or language.

Full texts of potentially eligible studies were then reviewed for inclusion based on the PICOS framework (Population, Intervention, Comparator, Outcomes, and Study Design), as outlined in Table 1.

**Table 1:** Eligibility criteria for inclusion in the systematic review according to the PICOS framework (Population, Intervention, Comparator, Outcomes, and Study Design).

Criterion	Inclusion	Exclusion
Population	Individuals diagnosed with cystic fibrosis	Individuals without cystic fibrosis; animal studies
Intervention or comparator	Any type of CFTR modulator therapy	Studies not reporting on CFTR modulators
Outcomes	Nasal endoscopy findings, nasal polyp score, or Lund‐Kennedy score	Studies not reporting relevant sinonasal outcomes
Study design	Original research articles including randomized controlled trials, non‐randomized controlled trials, single‐arm studies, cohort studies, case–control studies, cross‐sectional studies, registry/database studies, case reports	Clinical trial protocols, narrative reviews, editorials
Publication date	Studies published up to March 2025	Studies published after March 2025

Abbreviation: CFTR, cystic fibrosis transmembrane conductance regulator.

### 2.2 Data Collection

The following data were extracted from each study: author, year of publication, country of origin, study design, impact factor of the publishing journal, population characteristics, duration of follow‐up, type of cystic fibrosis gene and specific mutation, medication used and its therapeutic class, and nasal endoscopic outcomes assessed using standardized scoring systems. (Table 3).

For each study population, we specified the sex (male or female), age group (children or adults), and reported the mean age when available. Regarding treatment, each patient received the therapy most appropriate for their specific cystic fibrosis mutation. Although different nasal outcome scoring systems were employed across studies, a key inclusion criterion was that each study applied the same scale consistently before and after treatment, (Table 4) thereby ensuring valid intra‐study comparisons and reliable conclusions.

## 3 Results

**Figure 1:**
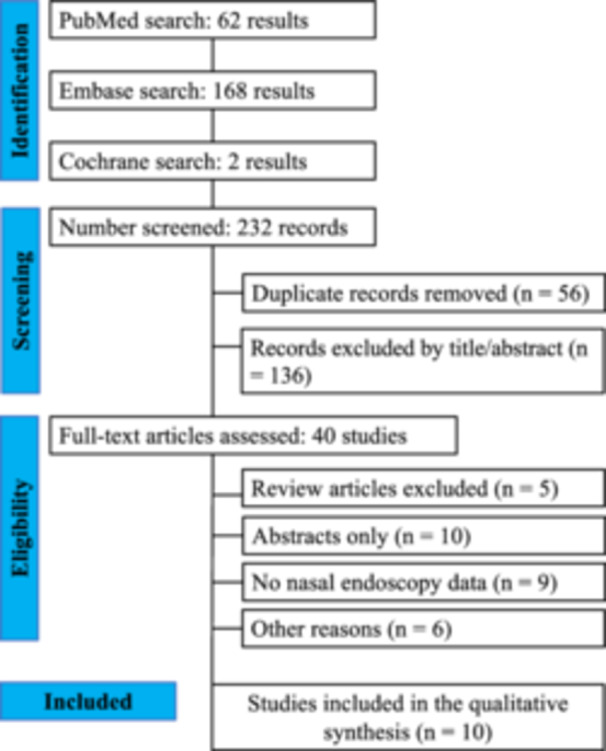
Preferred Reporting Items for Systematic Reviews and Meta‐Analyses (PRISMA) flow diagram of study screening and selection. N, number of studies. [Color figure can be viewed at wileyonlinelibrary.com]

From the 232 results initially identified, 56/232 (24.1%) were duplicate records and 136/232 (58.6%) were excluded based on title or abstract screening, leaving 40/232 (17.2%) studies for full‐text review. Among these, 10/40 (25%) had only the abstract available, 9/40 (22.5%) did not evaluate endoscopic findings, 6/40 (15.0%) fell into other exclusion criteria, and 5/40 (12.5%) were review articles. Ultimately, 10/40 (25.0%) studies met the inclusion criteria and were included in the final analysis (Figure 1).

Table 2 summarizes the studies selected for this systematic review, detailing the title of each article, the journal in which it was published, and the corresponding impact factor. Most studies evaluated the effects of CFTR modulators on sinonasal outcomes in patients with cystic fibrosis, covering different populations, clinical contexts, and types of modulator therapy.

**Table 2:** Characteristics of the studies included in the systematic review.

Study	Article title	Journal	Journal impact factor
Minzoni et al., 2024 [20]	Cystic fibrosis‐related chronic rhinosinusitis: The key role of a comprehensive evaluation in the era of highly effective modulator therapy	European Archives of Oto‐Rhino‐Laryngology and Head and Neck	1.9
Uyttebroek et al., 2024 [21]	Dual and triple modulator therapy for chronic rhinosinusitis in cystic fibrosis patients	Rhinology	6.8
Bode et al., 2023 [2]	Effects of *CFTR*‐modulator triple therapy on sinunasal symptoms in children and adults with cystic fibrosis	European Archives of Oto‐Rhino‐Laryngology and Head and Neck	1.9
Petit et al., 2025 [22]	Effects of Elexacaftor‐Tezacaftor‐Ivacaftor on nasal and sinus symptoms in children with cystic fibrosis	Pediatric Pulmonology	2.3
Di Gioia et al., 2024 [23]	Efficacy of Elexacaftor‐Tezacaftor‐Ivacaftor on chronic rhinosinusitis in cystic fibrosis	American Journal of Otolaryngology	1.7
Stapleton et al., 2022 [24]	Elexacaftor‐Tezacaftor‐Ivacaftor improves sinonasal outcomes in cystic fibrosis	Journal of Cystic Fibrosis	4.9
Stapleton et al., 2025 [7]	Elexacaftor‐Tezacaftor‐Ivacaftor improves sinonasal outcomes in young children with cystic fibrosis	International Forum of Allergy and Rhinology	6.8
Beswick et al., 2023 [25]	Factors that predict pursuing sinus surgery in the era of highly effective modulator therapy	International Forum of Allergy and Rhinology	6.8
Gostelie et al., 2020 [26]	The impact of ivacaftor on sinonasal pathology in S1251N‐mediated cystic fibrosis patients	PLOS One	2.9
Uyttebroek et al., 2023 [27]	Upper airway disease in adults with cystic fibrosis in the era of *CFTR* modulators	The Laryngoscope	2.0

Abbreviation: CFTR, cystic fibrosis transmembrane conductance regulator.

This systematic review comprised ten studies conducted across six countries: Germany, Belgium, France, Italy, the Netherlands, and the United States. The studies utilized various methodological designs, including retrospective cohort studies, prospective observational trials, and pre–post intervention evaluations. The included populations spanned both pediatric and adult age groups, with participants ranging from 5 to over 60 years of age. (Table 3) Follow‐up durations varied between 2 and 36 months. Across all studies, the majority of patients had moderate to severe cystic fibrosis, with a substantial proportion having previously undergone endoscopic sinus surgery.

**Table 3:** Description of the studies included in the systematic review.

Study	Country	Type of study	Population	Age	Follow‐up (months)	Pathogenic variants in the *CFTR* Gene	Treatment	Endoscopic nasal analysis
Minzoni et al., 2024 [20]	DE	Retrospective multicenter cohort	45 patients (25 women); 32 required prior surgery; mean BMI 20.9 ( ± 2.7); mean FEV_1_% 46.7 ( ± 19.9).	Mean 37.5 years ( ± 12.6)	12–14 months	F508del (12 homozygous, 33 heterozygous)	ELX/TEZ/IVA	Assessed by nasal polyp score (Meltzer) and modified Lund‐Kennedy score via nasal video endoscopy. Nasal polyp score: polyp size; modified Lund‐Kennedy score: polyp size, edema, secretion.
Uyttebroek et al., 2024 [21]	BE	Observational study	43 adult patients (26 men, 17 women); subgroups on ELX/TEZ/IVA (39) or TEZ/IVA (25); BMI ~ 23.5; mean FEV_1_% 73–77%.	Mean 32 years ( ± 9–10)	3 years, with 6–12 month intervals	F508del (70% homozygous, 30% heterozygous; some Class I/V pathogenic variants)	ELX/TEZ/IVA, TEZ/IVA	Rigid nasal endoscopy (4 mm, 30°); scored with Lund‐Kennedy (0–12) and modified Davos (0–8).
Bode et al., 2023 [2]	DE	Single‐center, cross‐sectional study	43 patients (25 women); mean baseline FEV_1_% 62.7. Control group: 20 patients (9 women), mean FEV_1_% 79.5.	Intervention: mean 32 years (14% < 18); Controls: mean 18 years (30% < 18)	Mean 9.3 months (2–16 months)	Intervention: F508del (53.5% homozygous, 46.5% heterozygous); Control: 30% homozygous, 70% heterozygous	ELX/TEZ/IVA	Endoscopy by same examiner; scored using nasal polyp score for polyp size.
Petit et al., 2025 [22]	FR	Prospective, single‐center, non‐randomized open‐label study	26 children aged 6–12; mean BMI 17.09 ( ± 3.11); 4 had prior sinus surgeries.	Mean 8.64 years (range 6–11.7)	12 months; baseline, 6 and 12 months assessments	86.4% F508del/F508del; 13.6% heterozygous (75% F508del/Minimal Function, 25% F508del/Residual Function)	ELX/TEZ/IVA	Polyps scored by LildHoldt and Johansson classification; mucosal hypertrophy scored by turbinate obstruction (0–2) bilaterally.
Di Gioia et al., 2024 [23]	IT	Retrospective cohort study	136 patients (66 men, 70 women); mean FEV_1_% 61.2% (20.7–114.3%); 26 (19.1%) had prior ESS; 28 underwent endoscopy.	Mean 32 years (13–56)	Mean 16.1 months (10–36)	50 (37.8%) F508del homozygous; 86 (63.2%) F508del heterozygous	ELX/TEZ/IVA	Endoscopic scores by Lund‐Kennedy scale (0–20); assesses polyps, secretion, edema, scarring, crusts.
Stapleton et al., 2022 [24]	US	Prospective, three‐center, pre–post study	34 patients (21 men); mean BMI 24.9; median FEV_1_% 80; 28 completed both visits.	Mean 28.9 years (12–60)	12 months; assessments every 6 months	19 (67.9%) F508del homozygous; 9 (32.1%) heterozygous	ELX/TEZ/IVA	Nasal endoscopy video recorded; scored by Lund‐Kennedy (0–2 per side for polyps, secretion, edema, scarring, crusts).
Stapleton et al., 2025 [7]	US	Prospective, three‐center, pre–post study	11 children (6 male, 5 female); 3 had prior ESS; 9 with endoscopy results.	Median 6.8 years (5–11.5)	Median 9 months	4 (36.4%) F508del/F508del; 7 (63.6%) F508del/Minimal Function	ELX/TEZ/IVA	Nasal endoscopy video recorded and scored with Lund‐Kennedy; median scores.
Beswick et al., 2023 [25]	US	Prospective, observational, multi‐institutional study	60 patients (63.3% female); mean FEV_1_% 75.6% (52.5–96.3%); > two‐thirds with prior ESS.	Mean 35.5 years ( ± 10.8)	12 months	F508del homozygous/heterozygous: 76.7%	ELX/TEZ/IVA	Endoscopy scored by Lund‐Kennedy (0–20).
Gostelie et al., 2020 [26]	NL	Prospective observational mono‐center cohort study	8 patients (5 male); 2 with prior ESS.	Mean 16 years (9–26)	Up to 12 months; mostly assessed at 2 months	All S1251N gating pathogenic variant (Class III); 7 F508/S1251N, 1 A455E/S1251N	IVA	Modified Lund‐Kennedy used for nasal polyps, discharge, and edema pre‐ and post‐Ivacaftor.
Uyttebroek et al., 2023 [27]	BE	Observational study	122 patients (71 men, 51 women); 53 on modulators, 69 controls.	Intervention: mean 31.1 years; Control: mean 34.13 years	Median 10 months	F508del homozygous (33); F508del heterozygous (19)	ELX/TEZ/IVA	Rigid nasal endoscopy (4 mm, 30°); Lund‐Kennedy for edema, polyps, secretion; polyp extent by modified Davos.

Abbreviations: BE, Belgium; BMI, body mass index; CFTR, cystic fibrosis transmembrane conductance regulator; DE, Germany (Deutschland); ELX, elexacaftor; ESS, endoscopic sinus surgery; FEV*1*, forced expiratory volume in 1 s; FR, France; IVA, ivacaftor; IT, Italy; NPS, nasal polyp score; NL, Netherlands; TEZ, tezacaftor; US, United States.

All participants carried pathogenic variants in the gene responsible for CFTR, with the F508del mutation appearing most frequently in both homozygous and heterozygous forms. The most commonly evaluated treatment was the combination of three modulator agents —Elexacaftor, Tezacaftor, and Ivacaftor — while others analyzed dual therapy (Tezacaftor and Ivacaftor) or monotherapy with Ivacaftor.

### 3.1 Sinonasal Assessment

Endoscopic evaluation of the upper airway was systematically performed using standardized tools such as the Lund‐Kennedy scoring system, nasal polyp grading scales, and modified instruments including the Davos and LildHoldt classifications. Across nearly all studies, the triple therapy showed consistent and clinically meaningful reductions in inflammation of the nasal and sinus cavities, as well as in the volume of secretions, edema, and size of nasal polyps (Table 4).

**Table 4:** Summary of clinical findings from studies on *CFTR* modulators in cystic fibrosis patients – sinonasal and pulmonary outcomes.

Study	Intervention	Before Treatment	After ELX/TEZ/IVA	Significant data	Other findings	Conclusion
Minzoni et al., 2024 [20]	ELX/TEZ/IVA	Before starting triple therapy, a comprehensive patient evaluation was conducted, including parameters relevant to nasal condition assessment. Nasal polyp score was recorded at 1.0, indicating small and localized nasal polyps, as assessed by nasal endoscopy. The mean Lund‐Kennedy score was 4.9.	After triple therapy, patient analysis revealed: nasal polyp score at 0.7, indicating mild polyp presence; mean Lund‐Kennedy at 2.4, reflecting a reduction in nasal and sinus pathologies, with decreased severity of polyps, secretions, and edema.	Patient scores showed a drop, especially in the Lund‐Kennedy average, indicating noticeable improvement in sinonasal conditions. Nasal polyp score reduction was mild but relevant.	The tools used — nasal polyp score and Lund‐Kennedy score — were effective in measuring nasal and sinus pathologies before and after triple therapy. Lung function improved (FEV_1_: 46.1 → 61.4%). Normosmia increased (26.7% → 77.8%). Olfactory recovery may have contributed to observed body mass index gain.	Triple therapy with ELX/TEZ/IVA demonstrated substantial effectiveness in alleviating sinonasal symptoms, improving pulmonary function and quality of life.
Uyttebroek et al., 2024 [21]	ELX/TEZ/IVA and TEZ/IVA	The mean Lund‐Kennedy score was 5.9 (SD ± 3) and Modified Davos score averaged 1.4 (SD ± 1.9).	After triple therapy: Lund‐Mackay index reduced by −1.6 (CI − 2.6 to −0.6); Modified Davos score reduced by −0.8 (CI − 1.4 to −0.1). After dual therapy: Lund‐Mackay index −0.7 (CI − 1.9 to 0.6); Modified Davos score −0.3 (CI − 1.4 to 0.8).	Patients receiving triple therapy showed significant reductions in sinonasal symptoms and endoscopic scores. Dual therapy also showed improvement, though less pronounced.	No other relevant findings reported in this article.	Triple therapy significantly improves sinonasal symptoms and reduces disease severity. Dual therapy was less effective but still showed benefit.
Bode et al., 2023 [2]	ELX/TEZ/IVA	Prior to triple therapy, patient conditions varied: polyps, mucosal swelling, or normal exams. Example: Patient 1 had left‐side polyps, right‐side swelling. Patients 3, 5, and 7 had moderate to severe polyps (nasal polyp score 3–4); Patients 2, 4, 6 showed normal findings.	After therapy: Most patients had no polyps; residual signs included mild erythema or small remaining lesions. Example: Patient 3 had no polyps with slight mucosal healing.	Substantial symptom reduction observed via nasal endoscopy.	Significant improvement in SNOT‐22 [Sino‐Nasal Outcome Test–22 (a symptom questionnaire)] scores, with mean decrease of 17 points after 9.3 months of therapy. All subdomains assessed showed clinical improvement.	Triple therapy showed clear benefits for sinonasal symptoms in patients, especially in reducing nasal polyps.
Petit et al., 2025 [22]	ELX/TEZ/IVA	Before treatment, 10 children (38%) had purulent secretion in the middle meatus. Nasal polyp score average was 0.58 with CI of [0; 1.6].	After evaluation, none of the children had purulent secretion. Nasal polyp score dropped to 0.23 [CI: 0–1.04], indicating a trend of improvement.	Nasal polyp score reduction wasn't statistically significant, but clinical trends and quality of life scores point toward improvement.	Antibiotic cycles decreased from 69 to 25 in the first treatment year. Turbinate hypertrophy dropped significantly by 1.4 points.	Triple therapy showed strong impact on nasal/sinus symptoms in children, reducing infection and enhancing quality of life. Trends suggest potential polyp regression over time.
Di Gioia et al., 2024 [23]	ELX/TEZ/IVA	Mean Lund‐Kennedy score before treatment was 4.21, with CI [3.38–5.05].	After therapy, the score decreased to 1.5, CI [1; 2], indicating significant improvement.	Paired t‐test confirmed statistical significance of the score reduction.	Recurrent sinusitis with chronic acute Episodes exacerbations dropped from 150 episodes to 62 across 136 patients; yearly average improved from 0.55 to 0.35 episodes.	The results indicate a substantial reduction in nasal/sinus compromise and a positive effect on patient quality of life.
Stapleton et al., 2022 [24]	ELX/TEZ/IVA	Prior to treatment, mean Lund‐Kennedy score was 6.0 [CI: 3–8.5], revealing considerable nasal and sinus involvement.	Post‐treatment, the score reduced to 2.0 [CI: 1–3.5], reflecting notable improvement.	Marked decline in endoscopic scores indicates effective clinical response.	Not reported	Triple therapy resulted in significant sinonasal improvement and enhanced patient quality of life.
Stapleton et al., 2025 [7]	ELX/TEZ/IVA	Lund‐Kennedy median score: 8; polyps in 89% of patients; crusts in 67%; secretion and edema in all patients.	Post‐treatment: Lund‐Kennedy median reduced to 4; polyps in only 11%; crusts resolved (0%). Secretion and edema persisted but were less viscous.	Polyps and crusts showed complete resolution following treatment.	Improved secretion viscosity noted, though not fully captured in Lund‐Kennedy grading system.	Endoscopic findings revealed near resolution of polyps and crusts. Remaining edema and secretion were less severe; therapy significantly enhanced quality of life.
Beswick et al., 2023 [25]	ELX/TEZ/IVA	Mean Lund‐Kennedy before treatment: 6.5 ± 4.9, with wide variability among patients.	Post‐treatment: score reduced to 5.2 ± 4.5, showing positive impact.	Score reduction considered clinically relevant despite modest numerical change.	Patients improved in appetite, weight gain, sleep quality, and Patient Health Questionnaire–9 Revised (for depressive symptoms) depression scores.	Treatment alleviated nasal obstruction and inflammation; improvement in comorbid symptoms supported better quality of life.
Gostelie et al., 2020 [26]	IVA	Before treatment: 50% had polyps only in the middle meatus; 50% had nasal discharge (thin and clear); 13% presented with mild edema; no thick purulent discharge reported.	After treatment: 88% had no polyps; 88% had no discharge; 50% had no edema; 38% had mild edema.	Clear improvement in sinonasal health and inflammation resolution.	Headaches resolved in 63% of patients post‐treatment versus 25% pre‐treatment; 1 patient showed olfactory recovery within 3 weeks.	Ivacaftor produced significant clinical improvements in nasal symptoms and contributed to reduced inflammation and improved wellbeing.
Uyttebroek et al., 2023 [27]	ELX/TEZ/IVA	Lund‐Kennedy score averaged 6/12, indicating moderate sinonasal inflammation pre‐treatment.	After treatment, score decreased to 4.9/12, indicating improvement.	Endoscopic improvement noted; score reduction reflects clinical benefit.	Bacterial colonization found in only 28% of treated patients versus 53% in untreated cohort.	CFTR modulators were effective in reducing nasal inflammation and bacterial colonization.

Abbreviations: CFTR, cystic fibrosis transmembrane conductance Regulator; CI confidence interval; ELX, elexacaftor; FEV_1_, forced expiratory volume in 1 s; IVA, ivacaftor (monotherapy); SD, standard deviation; TEZ, tezacaftor.

In the study by Minzoni et al. (2024), the average score on the Lund‐Kennedy scale decreased from 4.9 to 2.4, and the nasal polyp score declined from 1.0 to 0.7, indicating clear improvement [20]. Di Gioia et al. (2024) demonstrated a statistically significant reduction from 4.21 to 1.5 [23]. Similar results were reported in studies by Stapleton et al. (2025), with median scores dropping from 6.0 to 8.0 pre‐treatment to 2.0–4.0 after therapy [7]. Bode et al. (2023) described near‐complete polyp resolution in several cases [2].

In pediatric populations, such as the study conducted by Petit et al. (2025), purulent secretions resolved in all affected children and hypertrophy of the inferior turbinates diminished significantly [22]. Although nasal polyp score changes did not reach statistical significance in that study, the clinical outcomes were consistent with improved quality of life.

Dual therapy results were less pronounced. In the Belgian study, symptom and score reductions were smaller compared to the triple regimen, confirming the superior efficacy of three‐agent therapy [21]. Monotherapy with Ivacaftor also yielded benefits in a small sample, with notable reductions in nasal discharge and edema.

### 3.2 Pulmonary and Systemic Outcomes

Modulator therapy showed benefits that extended beyond the sinonasal region. Improvements in pulmonary function were reported, including increases in forced expiratory volume — particularly in studies conducted by Minzoni et al. (2024) and Beswick et al. (2024), with substantial gains documented after treatment [20, 25]. Stabilization of body mass and weight gain were also consistently observed (Table 4).

Olfactory recovery was frequently cited and associated with enhanced appetite and nutritional status, especially among pediatric patients. In some cases, improvements in sleep quality, emotional well‐being, and energy levels were also reported. For example, Beswick et al. identified reductions in depression scores and improved appetite, sleep, and overall vitality [25].

In addition, bacterial colonization of the nasal passages declined significantly in patients treated with modulators, as demonstrated in the Belgian study comparing treated and untreated groups [21]. Treated individuals showed a lower rate of colonization, suggesting an additional benefit related to infection control and mucosal immunity.

### 3.3 Clinical Instruments and Their Reliability

All scoring systems used across the studies demonstrated robust sensitivity and validity in identifying both baseline disease severity and improvements following therapy. The Modified Lund‐Kennedy scale proved effective in quantifying polyps, discharge, and edema, while complementary indices helped capture changes in mucosal appearance and patient‐reported outcomes.

### 3.4 Overall Interpretation

Taken together, the findings across these twelve studies strongly support the multifaceted benefits of modulator therapy targeting CFTR in the treatment of cystic fibrosis–associated sinonasal disease. The combination of three agents — Elexacaftor, Tezacaftor, and Ivacaftor — consistently demonstrated superior outcomes compared to dual or monotherapy.

The results highlight reductions in upper airway disease severity, improved pulmonary function, enhanced nutritional status, and better psychological well‐being. Although designs and follow‐up durations varied, the overall trend supports the routine integration of CFTR modulators into the management of both sinonasal and systemic manifestations of cystic fibrosis.

## 4 Discussion

CFTR modulator therapy has consistent and clinically meaningful benefits in reducing the global disease burden in patients with CF. However, this systematic review adds to the literature by synthesizing how effective these modulators are in improving sinonasal disease. These findings suggest that CFTR modulators offer a comprehensive therapeutic effect, addressing both local and systemic manifestations of the disease.

Among the ten studies included, the majority demonstrated improvement in endoscopic findings, such as reductions in nasal polyps, mucosal edema and purulent secretions, especially with the use of triple therapy combination, either in adults or children. This statement is consistent with the findings of Stapleton et al. (2025), whose study population ranged from childhood to adulthood [7]. Furthermore, the substantial heterogeneity among the study populations suggests that the improvements observed with CFTR modulators are not limited to specific patient profiles. All the studies analyzed included a heterogeneous population sample, with a wide age range and originating from different geographic regions. Considering that the clinical expression of cystic fibrosis can be influenced by environmental factors and genetic variability, this diversity adds robustness to the findings [28]. Even so, all the studies reported improvement in clinical outcomes after the therapeutic intervention.

The findings of this review align with and expand upon previous literature. Stapleton et al. (2025) reported that before triple therapy, 89% of the pediatric patients had nasal polyps according to Modified Lund‐Kennedy score (4,9), and after 9 months follow up, less than 11% had nasal polyps (4) [7]. Although the reduction in nasal polyp in children reported by Petit et al. (2025) was not statistically significant compared to Stapleton's study, this this difference was attributed to the use of a different nasal scoring system [7, 22]. While the first study used the Lund‐Kennedy score, the second used the LildHoldt & Johansson classification.

Minzoni et al. (2024), in their retrospective study evaluating only adults, reported that prior to triple therapy the mean Lund‐ Kennedy score was 4.9 [20]. In a long‐term follow‐up after treatment, the score decreased to 2.4, reflecting a global reduction in both nasal and sinus pathology [20]. Di Gioia et al. reported similar findings regarding the improvement in the Lund Kennedy score, which decreased from 4.21 before treatment to 1.5 after therapy [23].

Minzoni et al. (2024), Bode et al. (2023), Petit et al. (2025), and Di Gioia et al. (2024) exclusively applied triple therapy and observed significant clinical improvements, such as enhanced lung function, reduced nasal inflammation, and fewer respiratory infections [2, 20, 22, 23]. These findings were confirmed by clinical assessment tools, including endoscopy and laboratory tests, with statistically significant results. Regarding other symptoms beyond nasal polyps, Bode et al. (2023) reported a significant improvement in the total SNOT‐22 score (22‐item Sino‐Nasal Outcome Test), reflecting a reduction in ear, nose, and throat symptoms [2]. This is consistent with the findings of Petit et al. (2025), who noted the absence of purulent secretions after treatment, further supporting an improvement in patients’ quality of life [22].

The study by Uyttebroek et al. (2024) was the only one to directly compare double therapy (TEZ/IVA) with triple therapy (ELX/TEZ/IVA) [21]. Although both regimens provided clinical benefits, patients receiving triple therapy showed superior results, with greater symptom reduction and more favorable endoscopic scores. The study by Uyttebroek et al. (2023) evaluated double therapy alone and observed clinical improvement, but with less pronounced results compared to studies using triple therapy [27]. In other words, when dual therapy is compared with triple therapy, as in the study of Uyttebroek et al. (2024), both endoscopic scoring systems used (Lund‐Kennedy and modified Davos) showed significantly greater improvement with triple therapy. In conclusion, dual therapy found to be less effective [21].

The study by Gostelie et al. (2020) was the only one reporting results using Ivacaftor as monotherapy [26]. Before treatment, among the eight patients evaluated, 50% had polyps only in the middle meatus; 50% had nasal discharge (thin and clear); 13% presented with mild edema and no thick purulent discharge was reported. After therapy, 88% had no polyps; 88% had no discharge; 50% had no edema; and 38% had mild edema. To summarize, the medication also contributed to reducing inflammation and nasal symptoms [26].

The study has several limitations that should be considered when interpreting its findings. First, the number of studies specifically addressing sinonasal outcomes in patients with cystic fibrosis treated with CFTR modulators remains small. Many of the included studies had small sample sizes, especially in the pediatric population, and lacked randomized controlled designs, which may reduce the strength of the evidence. Additionally, some studies, such as those by Petit et al. (2025) and Gostelie et al. (2020), used different or non‐standardized endoscopic scoring systems, which limits the ability to make direct comparisons [22, 26]. The use of both the Lund‐Kennedy score and alternative scales such as the Lildholdt & Johansson or modified Davos classifications introduces variability in outcome reporting.

The heterogeneity observed in the results may be attributed to the presence of different genotypes of the *CFTR* gene in the cohorts, including both homozygous and heterozygous mutations, such as F508del and S1251N. Ideally, it would be possible to analyze the specific effect of these genotypes on sinonasal outcomes in response to CFTR modulators. However, it is important to note that none of the reviewed studies provided results stratified by genotype.

Nevertheless, the study by Minzoni et al. (2024) reported a cohort of 12 patients homozygous for the F508del mutation [20]. The results showed a significant reduction in the Nasal Polyp Score, from 1.0 to 0.7, and in the mean Lund‐Kennedy endoscopic score, from 4.9 to 2.4. This improvement not only demonstrates the effectiveness of the treatment but also suggests a strong response among homozygous patients. In Bode et al. (2023), which included 53.5% homozygous patients, a significant reduction in ear, nose, and throat symptoms and an improvement in the total SNOT‐22 score were observed, indicating that the therapy was effective in alleviating symptoms in a predominantly homozygous group [2].

In contrast, in Uyttebroek et al. (2024), where 30% of patients were heterozygous, the results were less pronounced compared to those seen in homozygous patients [21]. Although triple therapy showed effectiveness, the reduction in evaluation scores was smaller, reflecting a more modest response among heterozygous patients. In Petit et al. (2025), most patients (86.4%) were homozygous, and the results showed a significant improvement, with a reduced mean nasal polyp score and complete absence of purulent secretions [22]. This contrasts with studies that included a higher proportion of heterozygous patients, in which the improvements, although positive, did not reach the same magnitude.

Second, the heterogeneity across study populations and methodologies presents a challenge for data synthesis. Variations in age, baseline disease severity, prior surgical history, and follow‐up durations make it difficult to generalize findings to the broader cystic fibrosis population. Moreover, some studies only provided partial data or were available only as abstracts, limiting the depth of analysis. The retrospective nature of several studies also increases the risk of selection bias. Lastly, as CFTR modulator therapy is a rapidly evolving field, the inclusion of older studies may not fully reflect the latest developments or the long‐term impact of newer triple combination therapies.

The treatment duration also varied, ranging from 2 to 36 months, which may have influenced the effectiveness observed in each study. A longer treatment period was associated with more significant improvements, as seen in Uyttebroek et al. (2023) [27].

Future research should aim to address these limitations by conducting larger, multicenter, and prospective studies with standardized protocols for sinonasal assessment. The adoption of uniform scoring systems, such as the Lund‐Kennedy scale, across studies would enhance comparability and allow for meta‐analytical synthesis. In addition, more detailed subgroup analyses — stratifying patients by age, genotype, baseline sinonasal severity, and surgical history — would help identify which patient profiles benefit most from specific therapies, ultimately guiding personalized treatment approaches as well as long‐term follow‐up studies assessing the durability of sinonasal improvements.

## 5 Conclusion

CFTR modulator therapy represents a transformative advance for patients with cystic fibrosis. Beyond improving lung function and nutritional status, these agents offer measurable benefits for sinonasal inflammation and polyp burden. The consistent reductions in endoscopic scores highlight the added value of triple therapy. Standardizing sinonasal assessment tools will strengthen evidence comparability across future studies. Larger, prospective trials are needed to confirm long‐term outcomes. Personalized treatment plans based on genotype and baseline sinonasal severity should be encouraged. Clinicians should routinely integrate sinonasal monitoring into cystic fibrosis management. These findings support a holistic approach to optimize quality of life in cystic fibrosis care.

## Author Contributions

Rebecca Gallardo and Bruna Santos Silva collected and tabulated the data. Rebecca Gallardo Bruna Santos Silva interpreted the study findings. Rebecca Gallardo, Bruna Santos Silva, and Bruna Santos Silva, José Dirceu Ribeiro, and Eulalia Sakano wrote and revised the text thoroughly before submitting the manuscript to the scientific journal. Rebecca Gallardo, Bruna Santos Silva, Bruna Santos Silva, José Dirceu Ribeiro, and Eulalia Sakano approved the manuscript and agreed with its submission to the scientific journal.

## Funding

The authors have nothing to report.

## Ethics Statement

The authors have nothing to report.

## Conflicts of Interest

The authors declare no conflicts of interest.

## Data Availability

All information used in the research can be accessed upon reasonable request to the court's public records office.
